# Mechanisms of Polymyxin Resistance in Acid-Adapted Enteroinvasive *Escherichia coli* NCCP 13719 Revealed by Transcriptomics

**DOI:** 10.3390/microorganisms12122549

**Published:** 2024-12-11

**Authors:** Daekeun Hwang, Hyun Jung Kim

**Affiliations:** 1 Korea Food Research Institute, Wanju 55365, Republic of Korea; ant0040@korea.kr; 2 Department of Food Biotechnology, University of Science and Technology, Daejeon 34113, Republic of Korea

**Keywords:** *Escherichia coli*, acid adaptation, polymyxin resistance

## Abstract

Acid adaptation in *Escherichia coli* can induce antimicrobial resistance (AMR), posing challenges to global public health. We investigated the effects of acid adaptation on antimicrobial susceptibility, gene expression, zeta potential, and the outer membrane (OM) properties of *Escherichia coli* NCCP 13719. The acid-adapted (AA) strain exhibited increased resistance to multiple antimicrobials, with minimum inhibitory concentrations for colistin and polymyxin B increasing eight- and two-fold, respectively. Transcriptomic analysis identified 2225 differentially expressed genes, including upregulated genes associated with resistance to cationic antimicrobial peptides such as *arnCTE*, *marA*, and *tolC*. The upregulation of the *arn* operon suggests modifications in lipid A of lipopolysaccharides (LPS), reducing the negative charge of the OM and decreasing polymyxin binding affinity. Zeta potential measurements indicated a shift toward a less negative surface charge in the AA strain, which is consistent with LPS modifications. The AA strain also showed decreased OM permeability, which correlated with increased resistance to antimicrobials that penetrate the OM. These mechanisms collectively diminish the efficacy of polymyxins and highlight the potential for environmental factors to drive antimicrobial resistance. In conclusion, the acid adaptation of *E. coli* NCCP 13719 enhances AMR through changes in gene expression and OM modifications, highlighting the need for careful control of acidic environments during the treatment of medical devices and wastewater from food processing to prevent the emergence of resistant strains.

## 1. Introduction

Acid treatment is a widely used method for controlling or eliminating pathogenic microorganisms from the surfaces of medical devices, in wastewater treatment, and in the food industry [1,2,3]. The bactericidal mechanisms of acids include compromising the integrity of the outer membrane (OM), increasing the intracellular osmotic pressure, inhibiting macromolecular synthesis, depleting bacterial energy reserves, and inducing antibacterial peptide production in host cells [4]. However, inadequate acid treatment may lead to the emergence of acid-adapted (AA) microorganisms, which pose a significant challenge in the management of foodborne pathogens. Acid adaptation can induce a phenomenon known as cross-protection, where exposure to one stressor (acidic conditions) enhances resistance to other unrelated stressors (antimicrobial agents, temperatures, and salt) [5,6,7,8].

Antimicrobial resistance (AMR) represents a major global health challenge, with substantially increasing resistance to currently available antimicrobial agents against bacteria, fungi, viruses, and parasites [9]. The emergence of AMR among gram-negative bacteria is particularly alarming, as it has led to an increase in multidrug-resistant (MDR) organisms [9]. The increased antimicrobial resistance due to acid adaptation can lead to reduced efficacy of standard antibiotic treatments for *E. coli* infections. This necessitates higher doses or alternative antimicrobials, which may not always be available or could have more severe side effects [10]. Owing to the limited treatment options for MDR gram-negative bacteria, polymyxins have become crucial as a last resort treatment [11]. Polymyxins have been widely utilized for decades; however, resistance to polymyxins has arisen through multiple mechanisms. [12]. AMR is enhanced through various mechanisms including pumping the antimicrobial out of the cell, inactivating the drug using specialized enzymes, modifying the target structures to prevent interference, and bypassing the affected metabolic pathways [13].

As previously mentioned, AMR in foodborne pathogens can be induced by acid adaptation. In our previous study, polymyxin resistance was observed in AA pathogenic *Escherichia coli,* including ATCC 43889 (enterohemorrhagic *E. coli*) and NCCP 13717 (enterotoxigenic *E. coli*). The major mechanism of action was the upregulation of genes in the *arn* operon and lipopolysaccharide (LPS) modification [8,14]. Genes associated with polymyxin resistance have been well studied along with the *arn* operon [15,16].

Transcriptome analysis involves studying the entire set of RNA transcripts present in a cell or tissue at a specific time. By analyzing the transcriptome of the AA strain and comparing it to the transcriptomes of the control and relevant strains, differences in gene expression that may be associated with AMR can be identified. Through transcriptomic analysis, this study aimed to identify specific genes that were upregulated or downregulated in the AA strain compared with those in the control strain. This will allow us to explore the molecular mechanisms underlying AMR in the AA strain and gain insights into how changes in gene expression may contribute to its resistance phenotype.

## 2. Materials and Methods

### 2.1. Acid Adaptation of Pathogenic E. coli

The enteroinvasive *E. coli* strain NCCP 13719 (National Culture Collection for Pathogens, Osong, Republic of Korea) was subjected to acid adaptation, as previously described [14]. Acid adaptation was conducted under nutrient-rich conditions (AAR); the strain was cultured in tryptic soy broth (TSB; Merck, Darmstadt, Germany) adjusted to pH 5.0 and incubated at 37 °C for 100 h with constant agitation. After the exposure period, the surviving cells were spread onto tryptic soy agar (TSA; Merck, pH 7.2) and incubated at 37 °C for 24 h. Cultured colonies were considered to be those that survived at pH 5.0, and the pH of the TSB was incrementally reduced by 0.25 until colony recovery ceased. The minimum pH at which colony formation was observed was set to the pH to which the AA strain was adapted. Unadapted strains served as controls. All the bacterial cultures were stored at −80 °C in TSB containing 25% glycerol until further use.

### 2.2. Preparation of Inocula

Frozen stock cultures were streaked onto TSA plates and incubated at 37 °C for 24 h. A single well-isolated colony from each plate was then transferred to TSB (volume of 5 mL) and incubated at 37 °C for another 24 h. Two hundred µL of the AA *E. coli* culture was then inoculated into 5 mL of TSB adjusted to pH 3.5 and incubated at 37 °C for 50 h. A volume of 100 µL of the culture was then transferred to 10 mL of TSB (pH 7.2) and incubated at 37 °C for 24 h. Serial dilutions were made in saline to achieve an inoculum concentration of approximately 7 log CFU/mL. The prepared inoculum was used to evaluate the AMR (Section 2.3), acid survival (Section 2.4), zeta potential (ZP) (Section 2.7), and OM permeability (Section 2.8).

### 2.3. Antimicrobial Resistances

Antimicrobial susceptibility was evaluated using the broth microdilution method following EUCAST protocols [17], and the minimum inhibitory concentrations (MICs) of polymyxin B (PB) and colistin (CT) were evaluated. All the AMR data represent the results of three independent experiments, each of which was performed in duplicate.

### 2.4. Survival of Acid-Adapted E. coli in Acidic TSB

To evaluate the survival of acid-adapted *E. coli* in an acidic environment, 200 µL of the cell suspension prepared as described in Section 2.2 was inoculated into 5 mL of fresh acidic TSB adjusted to pH 3.5, as outlined in our earlier study [14]. The cultures were incubated at 37 °C with agitation for 24 h. After incubation, the samples were serially diluted in saline and spread onto MacConkey agar plates (Thermo Fisher Scientific, Waltham, MA, USA). The plates were incubated at 37 °C for 24 h, and colonies were enumerated to determine the viable bacterial concentration, expressed as log CFU/mL.

### 2.5. Transcriptomic Analysis

Gene expression comparisons between the AAR and control strains were performed using transcriptomic analysis. The total RNA was isolated, and libraries were prepared as outlined in our earlier study [8]. Briefly, RNA was extracted using the Qiazol lysis reagent (Qiagen GmbH, Hilden, Germany), followed by DNA removal using on-column RNase-Free DNase I (Qiagen). After RNA purification, the RNA was depleted using the Ribo-Zero rRNA Removal Kit (Illumina, San Diego, CA, USA). Sequencing was carried out using the Illumina HiSeq × 10 platform with 150 bp paired-end reads. Transcriptome data were collected from three biological replicates.

Raw RNA-seq data were processed as described previously [14] using OmicsBox software (v3.0) [18]. Quality control and read trimming were performed using FastQC (v0.11.9) for quality assessment and Trimmomatic (v0.38) for trimming, removing low-quality reads (Q > 20) and excluding adapters. De novo transcriptome assembly was conducted using the Trinity assembler package (v2.15.1). Sequence alignment against the NCCP 13719 draft genome was performed using Bowtie2 (v2.4.2), and gene read counts were determined using HTSeq (v0.9.1). Differentially expressed genes (DEGs) were identified using EdgeR (v3.28.0) using the trimmed mean of M-values (TMM) method. Significant differences were defined as those with a false discovery rate (FDR) < 0.05 and a minimum two-fold change. DEG expression levels were reported as a log2 fold change (FC).

Kyoto Encyclopedia of Genes and Genomes (KEGG) pathway analysis was performed to identify the functional enrichment of DEGs. DEG lists were submitted to the ShinyGO v0.80 web application (http://bioinformatics.sdstate.edu/go80/, accessed on 27 November 2024) for KEGG pathway evaluation [19].

### 2.6. qRT-PCR Validation

Quantitative real-time PCR (qRT-PCR) was conducted to validate the RNA-seq results [8]. The expression levels obtained from qRT-PCR were normalized against housekeeping genes (*16S* rRNA) to account for any variations in RNA quantity and quality. Primers for target genes and the *16S* rRNA housekeeping gene were designed using Primer3 v4.1.0 (https://bioinfo.ut.ee/primer3/, accessed on 2 June 2024) and a previous report [14]. The thermal cycling conditions are listed in Table S1. The 2^−ΔΔCt^ method was used to quantify the relative expression levels of each gene, normalized to the *16S* rRNA internal control [20]. All the results are representative of four independent experiments conducted in duplicate.

### 2.7. Zeta Potential Measurements

The ZP is defined as the effective electrostatic potential at the interface. The membrane potential was measured using a ZP analyzer. The procedure described by Gogry et al. [21] was used [21]. As described in Section 2.2, the prepared bacterial inoculum was washed twice with deionized water (DW) and resuspended in DW at a concentration of 10^7^ CFU/mL. The bacterial suspensions were loaded into disposable folded capillary cells (Malvern, Worcestershire, UK). The ZP of the bacterial cells was measured at 25 °C with an applied voltage of 150 V using a ZP analyzer (Analyzer Malvern Zeta sizer Nano ZS, Malvern). All the data represent the results of two independent experiments, each conducted in triplicate.

### 2.8. OM Permeability

The OM permeability was evaluated using 1-N-phenylnaphthylamine (NPN) uptake [22]. The cells prepared according to Section 2.3 were collected by centrifugation and resuspended in a buffer containing 5 mM HEPES and 5 mM glucose (pH 7.4). The bacterial suspensions were adjusted to A600 = 1.0 and incubated with NPN (3.0 µmol/L) at 37 °C for 30 min in the dark. The fluorescence intensity was measured using a SpectraMax i3x microplate reader (Molecular Devices, San Jose, CA, USA) with excitation and emission wavelengths set at 350 nm and 450 nm, respectively. The NPN uptake was determined using the following Equation (1):

Relative fluorescence intensity (%) = F_1_/F_0_ × 100 (1)

where F_0_ is the fluorescence intensity of the untreated control cells, and F_1_ is the intensity of the AAR cells. The data represent the results of three independent experiments, each conducted in triplicate.

### 2.9. Statistical Analyses

All the statistical analyses were conducted using GraphPad Prism 9 software (Boston, MA, USA). The results are presented as the mean ± standard deviation with the statistical significance set at *p* < 0.05. Unpaired t-tests were employed to determine statistical differences in acid survival, zeta potential, and outer membrane permeability between the control and AAR strains.

## 3. Results and Discussion

### 3.1. Altered AMR

The AMR patterns of eight *E. coli* strains, including seven pathogenic and one commensal strain, after long-term acid adaptation were reported in our previous study [14]. Among the strains tested, both NCCP 13719 and 13717 showed enhanced resistance to polymyxins (CT and PB), and we further tested the MIC of strain NCCP 13719 against polymyxins in this study (Table 1). We also enriched the WHO classifications to provide a more detailed interpretation of the strain resistance profile and its potential clinical implications. While the NCCP 13719 control strain was susceptible to all the tested antimicrobials, the NCCP 13719 AAR strain showed increased resistance to several antimicrobials compared with that of the control. These include ampicillin, amoxicillin/clavulanic acid, and ampicillin/sulbactam, which are classified as highly important antimicrobials, as well as gentamicin, which is a critically important antimicrobial. Additionally, the AAR strain demonstrated increased resistance to cefotaxime, ciprofloxacin, CT, and PB, all of which were classified as highest-priority critically important antimicrobials (Table 1). The increased AMR of the AAR strain suggests that a stressful environment may have triggered mechanisms, such as efflux pump activation, OM changes, or the expression of specific resistance genes that enhance survival. This shift is particularly concerning because resistance to these antimicrobials can compromise clinical treatment options, especially for infections involving MDR gram-negative pathogens. In the absence of acid adaptation in the control, the bacterial strain did not display resistance, indicating that the resistance was induced rather than intrinsic.

**Figure 1:**
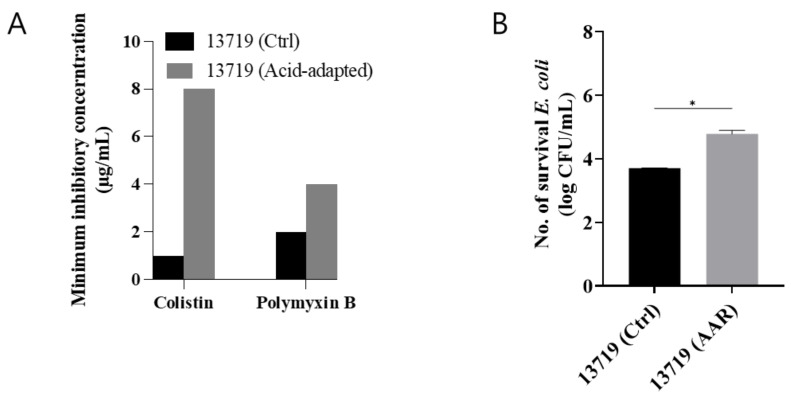
Phenotypic alterations resulting from acid adaptation with rich nutrients. (**A**) Minimum inhibitory concentration differences of polymyxins between NCCP 13719 control and NCCP 13719 AAR strains. (**B**) Viable *E. coli* count under low pH (pH 3.5) post 24 h cultivation in tryptic soy broth at 37 °C. Statistical significance was determined using an unpaired *t*-test: *p* < 0.05 (*).

The increased polymyxin resistance of *E. coli* observed in this study after acid adaptation is consistent with previous studies [8,14,23]. As per the EUCAST guidelines [24], polymyxin resistance is characterized by MIC > 2 μg/mL, while susceptibility is defined as MICs ≤ 2 μg/mL. The MICs for CT and PB in the NCCP 13719 control strain were 1 and 2 μg/mL, respectively. In contrast, the NCCP 13719 AAR strain exhibited an eight-fold increase in MIC for CT and a two-fold increase for PB, reaching 8 and 4 μg/mL, respectively (Figure 1A and Table 1).

Although resistance to polymyxins was observed in both the 13717 and 13719 strains [14], the MIC changes were different in these strains. In this study, we observed a significant increase in resistance to PB and a relatively small increase in resistance to CT for strain 13719, whereas for strain 13717, we observed a four-fold increase in MIC for PB and a two-fold increase in MIC for CT. Most gram-negative pathogens, including *Listeria monocytogenes*, *Salmonella enterica*, *Acinetobacter baumannii*, and *Cronobacter sakazaki* become resistant to multiple antimicrobials when exposed to acid [5].

### 3.2. Survival in Acidic Medium

In addition to the polymyxin resistance of the NCCP 13719 AAR strain, we compared the survival of the NCCP 13719 AAR and NCCP 13719 control strains in an acidic medium. When the cells were incubated in TSB (pH 3.5) for 24 h, the survived cell count of NCCP 13719 AAR was 4.8 ± 0.1 log CFU/mL, and the NCCP 13719 control was 3.7 ± 0.0 under the same conditions (*p* < 0.05, Figure 1B). Compared with the control, the NCCP 13719 AAR strain exhibited enhanced survival in an acidic environment. Consistent with this, former reports have shown that AA *E. coli* O157:H7 has increased survivability in acidic environments compared to non-adapted *E. coli* [6,14,25,26,27,28].

### 3.3. Analysis of DEGs and Functional Analysis

We found that the NCCP 13719 strain demonstrated enhanced resistance to polymyxins (CT and PB) and survival in acidic media compared with that of the control. We hypothesized that specific genes were involved in the increased resistance and survival of the 13719 AAR strain. Therefore, we analyzed the DEGs of the 13719 AAR strain to identify the specific genes responsible for determining resistance to PB and CT and survival in acidic environments.

RNA was extracted from two strains (NCCP 13719 control and AAR strain) that had reached the stationary phase after incubation for 24 h and was used for analysis. The total number of DEGs between NCCP 13719 AAR and the NCCP 13719 control cells, determined using the criteria of |log_2_FC| ≥ 1 and FDR < 0.05, was 2225, comprising 543 upregulated genes and 1682 downregulated genes.

**Figure 2:**
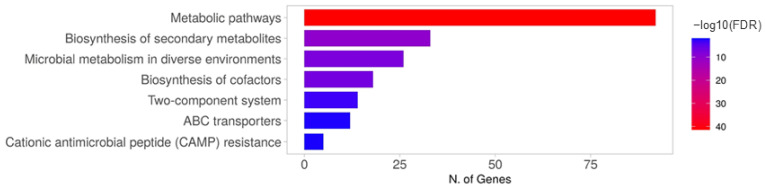
Kyoto Encyclopedia of Genes and Genomes pathway enrichment analysis for upregulated genes between control vs. acid adaptation with rich nutrients.

To further investigate the biological pathways associated with NCCP 13719 AAR, the DEGs were mapped to KEGG pathways, with a significance level of *p* < 0.05. Subsequent classification of the KEGG pathways for the upregulated genes revealed that DEGs were predominantly enriched in pathways associated with metabolism, the biosynthesis of secondary metabolites, microbial metabolism in diverse environments, amino acid biosynthesis, and a two-component system (Figure 2). These findings indicate that exposure to sublethal acid concentrations primarily affects cellular metabolism and the associated pathways. The analysis revealed several upregulated genes associated with CAMP resistance, including *arnCTE*, marA, and *tolC* (Figure S1).

**Figure 3:**
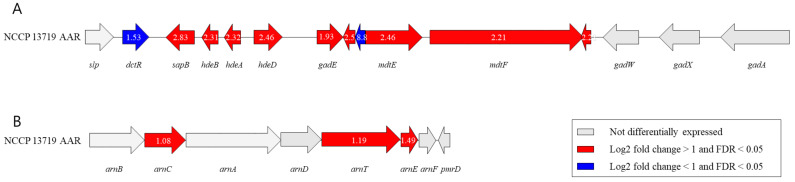
Genomic arrangement of genes linked to the acid-fitness island and the *arn* operon. Arrows, scaled to represent gene length and orientation, illustrate the log_2_ fold change in upregulation for each gene based on RNA-seq analysis. (**A**) Acid-fitness island and (**B**) *arn* operon.

The classification of the DEGs observed in the NCCP 13719 AAR strain into stress-, acid stress-, and polymyxin resistance-related genes is shown in Table 2; the significantly upregulated DEGs were *ompXW*, *sapB*, *mdtE*, and *hdeD*. Table S2 lists the top 50 upregulated and 50 downregulated DEGs. The genomic organization of genes implicated in the acid stress response, including the acid-fitness island (AFI) and the *arn* operon, is illustrated in Figure 3. Both upregulated and downregulated genes were observed in the AFI of the NCCP 13719 AAR strain. Log_2_ fold change values, as determined by RNA-seq analysis, were annotated within each arrow to quantify gene expression alterations. Most of the genes in the AFI were upregulated by up to 2.83 log_2_ fold. Relatively low fold changes (1.08–1.49 log_2_ fold changes) were observed for genes in the *arn* operon.

As shown in Table 2 and Figure 3A, most AFI genes, such as *sapB*, *hdeBAD*, *gadE*, and *mdtEF*, were upregulated with fold increases of 2.83, 2.31, 2.32, 2.46, 1.93, 2.46, and 2.21, respectively. Only two genes were downregulated in AFI.

Acidic extracellular stress results in cytoplasmic acidification, which in turn activates *EvgA* and, subsequently, *tolC*, a crucial element of the MDR efflux pumps in *E. coli*. This was followed by increased expression across various strains, as corroborated by Du et al. [29]. In NCCP 13719 AAR, *tolC* was 1.05-fold upregulated (Table 2). The essential role of tolC in the expression of *gadA* and *gadB* is well-documented [30], i.e., the importance of the interaction between *tolC* and *gadBCE*, which is critical for bacterial survival in acidic and antimicrobial-rich environments. In addition, the marked upregulation of the gad operon may play a role in antimicrobial resistance (AMR) by increasing the expression of *tolC* and *mdtEF*. The *mdtE* and *mdtF* genes exhibited significant changes in NCCP 13719 AAR cells, with 2.46-fold and 2.21-fold increases, respectively. (Table 2 and Figure 3A).

Given that polymyxin resistance was phenotypically observed, DEGs related to polymyxin resistance were also identified. Genes associated with polymyxin resistance have been well studied along with the *arn* operon, which collectively modifies LPS by the addition of 4-amino-4-deoxy-L-arabinose (L-Ara4N), leading to a decrease in binding affinity with polymyxins [15,16]. In the NCCP 13719 strain, AAR adaptation led to the upregulation of *arnCTE* genes, with fold increases of 1.08, 1.19, and 1.49 (Table 2 and Figure 3B). However, the patterns of upregulated genes in the *arn* operon were different for each pathogenic *E. coli*. In addition to strain NCCP 13719 AAR (*arnCTE* genes upregulated in enteroinvasive *E. coli*), the upregulation of *arn* genes has also been reported in other pathogenic *E. coli* strains, including ATCC 43889 (*arnA* gene upregulated in enterohemorrhagic *E. coli*) and NCCP 13717 (*arnBCAD* genes in enterotoxigenic *E. coli*) [8,14]. Notably, the strains exhibiting polymyxin resistance had one or more upregulated genes within the *arn* operon. Although the upregulation of the *arn* operon may be an important mechanism of polymyxin resistance in AA pathogenic *E. coli*, further studies on other pathogenic *E. coli* are warranted to clarify the mechanisms of polymyxin resistance in pathogens. Given the observed variations in *arn* operon expression among pathogenic *E. coli* strains with distinct virulence gene profiles, it is essential to investigate the mechanisms underlying polymyxin resistance in strains with diverse virulence characteristics.

Several studies have shown that *arnA* gene expression is significantly higher in polymyxin-resistant strains compared to polymyxin-susceptible strains [8,14,31]. Similar to the results of this study, the *arnT* gene was found to be significantly upregulated in the resistant strain, which was explained by altered L-Ara4N fixation to lipid A [32]. Regarding the importance of the *arnT* gene, it has been reported that deletion of the *arnT* gene, which removes L-Ara4N from lipid A, alters the overall charge of the molecule, increasing susceptibility to PB [33]. These findings, along with the data from the present study, demonstrate that polymyxin-resistant strains exhibit distinct gene expression profiles and upregulation within the *arn* operon can enhance polymyxin resistance.

Other genes involved in polymyxin resistance have also been identified. Groisman et al. suggested that low pH and low Mg^2+^ concentrations may induce the expression of the *PmrA* promoter in phagocytes, thereby promoting resistance to polymyxins [34]. A recent study identified the *RpoE* stress response pathway as crucial for polymyxin resistance in *E. coli*, highlighting mutations in *rseP*, *degS*, and *surA* that increase the susceptibility to CT [35]. The mobile CT resistance gene (*mcr*) has been suggested as another polymyxin resistance mechanism [36]. However, *mcr* genes were not found among the DEGs in the NCCP 13719 AAR strain in this study. Similarly, gram-negative bacteria isolated from multiple sources showed polymyxin resistance, although they lacked *mcr* genes [37]. Previous studies revealed that acid adaptation upregulates the *arn* operon and increases resistance to polymyxins without the participation of *mcr* genes [5,8,14]. Therefore, polymyxin resistance may be induced by incomplete acid treatment, even in the absence of *mcr* genes.

### 3.4. Validation of Transcriptomic Analysis

To validate the RNA-seq data, qRT-PCR was performed on the selected DEGs. These genes were chosen because of their pronounced differential expression as observed in the RNA-seq data (Table 2). The qRT-PCR results, expressed as mean values with standard deviations, strongly agreed with the RNA-seq data for all the selected DEGs (Figure S2). The concordance confirms that the differential gene expression identified by RNA-seq is reliable. It validates the accuracy of the RNA-seq technology in detecting true changes in gene expression levels under AA conditions.

### 3.5. ZP Measurements

**Figure 4:**
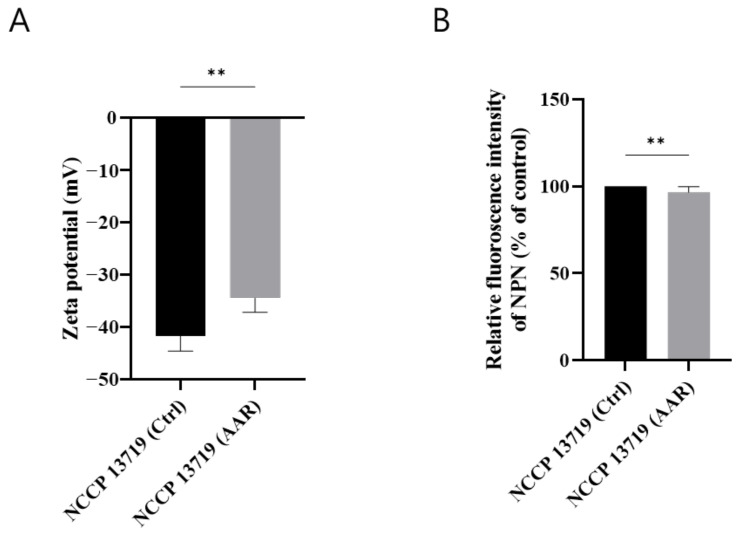
Evaluation of cell surface charge and outer membrane permeability in control and acid-adapted strains. (**A**) Cell surface charge based on the zeta potential of *E. coli*. (**B**) Outer membrane permeability assessed by 1-N-phenylnaphthylamine (NPN) uptake in *E. coli*. Statistical significance was determined by unpaired *t*-test: *p* < 0.01 (**).

The ZP of *E. coli* strain NCCP 13719 under control and AAR conditions was assessed to provide insights into the surface charge characteristics that influence bacterial interactions with their environment and antimicrobial agents. The control strain displayed a ZP of approximately −41 mV (Figure 4A). The observed negative ZP *E. coli* cells in the stationary phase were comparable to those seen in other gram-negative bacteria under similar conditions (around −40 mV) [21,38]. After AAR, this strain also showed a positive increase in ZP to approximately −34 mV in the AAR strain (Figure 4A). These changes suggest modifications in cell surface properties due to AAR and the upregulated *arn* operon, and the increase in the ZP could potentially affect electrostatic interactions with the environment and antimicrobials, such as polymyxins. Previous studies have shown similar findings to ours; among gram-negative pathogens, polymyxin-resistant strains had increased ZP compared with that of non-resistant strains [39,40,41,42]. The pattern of increased positive charge across gram-negative strains suggests the possibility of mechanisms that may alter cell-surface dynamics to enhance survival or resistance under stress conditions [15,31,32].

### 3.6. OM Permeability

We found that the OM-related genes were upregulated (Table 2). In order to determine whether gene expression affected OM changes in AAR cells, OM permeability was evaluated. The OM permeability was found to be approximately 10% lower in the AAR cells than in the control cells (*p* < 0.0001, Figure 4B). Strains undergoing AAR adaptation exhibited a marked reduction in OM permeability, suggesting the possible reinforcement of the OM as a protective response to acidic stress. This decrease in permeability may hinder the entry of acids and antimicrobial agents, potentially contributing to the resistance observed in these strains.

The relevance of these observations is highlighted in the existing literature, which associates reduced OM permeability in gram-negative bacteria with increased AMR. Diminished permeability serves as a physical barrier that restricts the penetration of antimicrobial compounds, thus reducing bacterial susceptibility [43,44]. The channel size was decreased under acid exposure, which could impact susceptibility to various antimicrobials, including β-lactams, aminoglycosides, macrolides, rifamycins, novobiocin, fusidic acid, and polymyxins [43]. The NCCP 13719 AAR cells showed increased resistance to amoxicillin/clavulanic acid, ampicillin/sulbactam, gentamycin, and polymyxins compared to the control (Table 1). Furthermore, the OM permeability results suggested that acid adaptation induces modifications in the bacterial cell envelope, particularly affecting the barrier function of the OM.

Furthermore, we observed upregulation of the *ompX* gene in the acid-adapted strain. *OmpX* encodes a porin with an eight-stranded β-barrel structure similar to *OmpA*. The overexpression of *ompX* has been associated with decreased levels of other porins, such as Omp36, which can impede the penetration of antibiotics like polymyxins [45]. The *ompX* expression is 1.7-fold higher in antimicrobial-resistant S. Typhi isolates than in antimicrobial-sensitive isolates, which is consistent with our results [46]. While our results highlight a potential link between OM permeability alterations and AMR induced by acid adaptation, further studies are needed to clarify the exact molecular mechanisms involved. Direct experimental evidence, such as measuring the changes in specific porin levels and assessing their impact on antibiotic uptake, would strengthen the understanding of this relationship.

Further research, including whole-genome sequencing and comparative genomics between the acid-adapted (AA) and control strains, is needed to identify gene mutations.

## 4. Conclusions

Acid adaptation of enteroinvasive *E. coli* NCCP 13719 significantly enhanced AMR, particularly against polymyxins, through multifaceted mechanisms involving alterations in gene expression and OM properties. Acid adaptation exhibited increased MICs for CT and PB, with eight- and two-fold increases, respectively, compared with those of the control. Transcriptomic analysis revealed the upregulation of genes involved in acid stress and CAMP resistance, including *arnCTE*, *marA*, and *tolC*. Genes in the *arn* operon were upregulated, but the patterns differed from those in AA enterohemorrhagic *E. coli* (ATCC 43889) and enterotoxigenic *E. coli* (NCCP 13717), suggesting differences in specific regulation. Upregulation of the *arn* operon suggests the modification of lipid A in LPS, reducing the net negative charge of the OM and decreasing the binding affinity for polymyxins. ZP measurements corroborated these findings, showing a shift toward a less negative surface charge in the AA strain. Additionally, decreased OM permeability was observed in the AA strain, likely due to the upregulation of *ompX*, leading to reduced porin size and limited antimicrobial penetration. Decreased permeability is correlated with increased resistance to antimicrobials, including β-lactams and aminoglycosides, which rely on OM penetration. Considering differential gene expression in pathogenic *E. coli* strains with different toxigenic potentials, it is important to understand the mechanisms underlying polymyxin resistance in different pathogenic *E. coli* strains. The results of this study underscore the potential risk of polymyxin resistance in AA pathogenic *E. coli*. Therefore, infection control measures should be strengthened, for example, by avoiding sterile processes under sublethal acid conditions to mitigate the risk of AA strains occurring in medical and wastewater treatment and the food industry. Understanding the molecular mechanisms underlying acid-induced AMR is crucial for developing strategies to mitigate the emergence and spread of AMR bacterial strains.

## Figures and Tables

**Table 1:** Details of the antimicrobial agents tested and antimicrobial resistance profile of enteroinvasive *E. coli* NCCP 13719.

	Antimicrobials		Disk Contents (μg)	WHO Classifications ^1^	Antimicrobial Resistance of NCCP 13719 Strain		Reference
Category	Abbreviations	Full Name			Control	Acid-Adapted	
Penicillins	AMP	Ampicillin	10	HIA	S	R	[14]
	AMC	Amoxicillin/Clavulanic Acid	30	HIA	S	R	[14]
	SAM	Ampicillin/Sulbactam	20	HIA	S	R	[14]
Aminoglycosides	CN	Gentamicin	10	CIA	S	R	[14]
Carbapenems	IPM	Imipenem	10	Authorized for use in humans only	S	S	[14]
Sulfonamide	SXT	Sulfamethoxazole/Trimethoprim	25	HIA	S	S	[14]
Cephalosporins	FEP	Cefepime (4th)	30	HP-CIA	S	S	[14]
	CTX	Cefotaxime (3rd)	30	HP-CIA	S	I	[14]
	CRO	Ceftriaxone (3rd)	30	HP-CIA	S	S	[14]
	FOX	Cefoxitin (2nd)	30	HIA	S	S	[14]
Phenicols	C	Chloramphenicol	30	HIA	S	S	[14]
Quinolones	CIP	Ciprofloxacin	5	HP-CIA	S	I	[14]
			MIC of ECOFFs ^2^ (μg/mL)		MIC of NCCP 13719 strain against polymyxins (μg/mL)		Reference
Category	Abbreviations	Full name		WHO Classifications	Control	Acid-adapted	
Polymyxins	CT	Colistin	2	HP-CIA	1 (S)	8 (R)	This study
	PB	Polymyxin B	2	HP-CIA	2 (S)	4 (R)	This study

^1^ WHO: World Health Organization; CIA: critically important antimicrobial; HIA: highly important antimicrobial; HP-CIA: highest-priority critically important antimicrobial. ^2^ ECOFFs: EUCAST epidemiological cutoff values (Report resistant (R) for isolates with MIC above the cut-off value. Otherwise report susceptible (S)).

**Table 2:** Selected differentially expressed genes (DEGs) associated with stress response and polymyxin resistance.

Gene	Product	Log_2_ Fold Change *	FDR **
Stress-related gene			
*uspA*	Universal stress protein A	−2.11	1.60 × 10^−13^
*uspD*	Universal stress protein D	1.22	3.03 × 10^−11^
*uspF*	Universal stress protein F	−3.23	3.21 × 10^−106^
*cspA*	Cold shock protein CspA	−1.00	3.98 × 10^−1^
*cspG*	Cold shock-like protein CspG	−3.65	9.18 × 10^−3^
*cspC*	Cold shock-like protein CspC	−2.18	1.50 × 10^−2^
*rpoS*	RNA polymerase sigma factor RpoS	−10.23	3.63 × 10^−125^
*ftsP*	Cell division protein FtsP	−1.29	4.75 × 10^−4^
*yaaA*	Peroxide stress resistance protein YaaA	1.27	2.04 × 10^−8^
Acid stress-related gene			
*dctR*	HTH-type transcriptional regulator DctR	−1.53	1.56 × 10^−30^
*sapB*	hypothetical protein	2.83	2.09 × 10^−11^
*hdeB*	Acid stress chaperone HdeB	2.31	1.58 × 10^−4^
*hdeA*	Acid stress chaperone HdeA	2.32	1.68 × 10^−4^
*hdeD*	Protein HdeD	2.46	4.97 × 10^−6^
*gadE*	Transcriptional regulator GadE	1.93	1.08 × 10^−7^
*mdtE*	Multidrug resistance protein MdtE	2.46	1.55 × 10^−42^
*mdtF*	Multidrug resistance protein MdtF	2.21	5.90 × 10^−178^
Polymyxin resistance-related gene			
*arnC*	Undecaprenyl-phosphate 4-deoxy-4-formamido-L-arabinose transferase	1.08	1.84 × 10^−2^
*arnT*	Undecaprenyl phosphate-alpha-4-amino-4-deoxy-L-arabinose arabinosyl transferase	1.19	8.26 × 10^−7^
*arnE*	putative 4-amino-4-deoxy-L-arabinose-phosphoundecaprenol flippase subunit ArnE	1.49	1.45 × 10^−2^
*eptB*	Kdo(2)-lipid A phosphoethanolamine 7″-transferase	−1.79	3.13 × 10^−16^
*ugd*	UDP-glucose 6-dehydrogenase	−7.46	5.06 × 10^−29^
*ompX*	Outer membrane protein X	3.32	1.17 × 10^−19^
*ompW*	Outer membrane protein W	2.90	5.85 × 10^−5^
*ompN*	Outer membrane porin N	1.75	7.87 × 10^−6^
*tolC*	Outer membrane protein TolC	1.05	2.73 × 10^−10^

* Blue color indicates down-regulated genes and red color indicates up-regulated genes. ** FDR: false discovery rate.

## Data Availability

The RNA-seq data have been deposited in the GenBank database (accession numbers SAMN41009044 and SAMN41009045).

## Supplementary Materials

The following supporting information can be downloaded at https://www.mdpi.com/article/10.3390/microorganisms12122549/s1, Figure S1: Identification of upregulated genes associated with cationic antimicrobial peptide resistance using KEGG pathways; Figure S2. Validation of RNA-seq results via qRT-PCR. Expression level comparison of 10 selected DEGs between RNA-Seq and qRT-PCR; Table S1: Primer sequence for qRT-PCR; Table S2. List of top 50 differentially expressed genes (DEGs).
