# Supplementary material for: Mechanisms of Polymyxin Resistance in Acid-Adapted Enteroinvasive Escherichia coli NCCP 13719 Revealed by Transcriptomics
Source: Microorganisms. 2024 Dec 11;12(12):2549. doi: 10.3390/microorganisms12122549 (PMC11677122; doi:10.3390/microorganisms12122549)
Supplement: Supplementary file 1 [file microorganisms-12-02549-s001.zip › microorganisms-3351165-supplementary.pdf]

Supplementary data

Mechanisms of Polymyxin Resistance in Acid-adapted Enteroinvasive *Escherichia coli* NCCP 13719 Revealed by Transcriptomics

Daekeun Hwang[a,b], Hyun Jung Kim[a,b*]

[a] Korea Food Research Institute, Wanju, Jeolla-buk, 55365, Korea

[b] Department of Food Biotechnology, University of Science and Technology, Daejeon 34113, Korea

*Corresponding author: Tel.: +82 63 219 9271; Fax: +82 63 219 9876; E-mail: hjkim@kfri.re.kr

Supplementary data

Table S1. Primer sequence for qRT-PCR.

Gene	Sense primer sequence (5′ → 3′)	Antisense primer sequence (5′ → 3′)	References
rpoS	CGAATAGTACGGGTTTGG	CGTTGCTGGACCTTATC	(Kay et al., 2017)
ftsP	TGATTTCAGTTGGCAGCAAG	ACGCCAGAATACAACGAACC	(Hwang & Kim, 2024)
gadE	CGCTCAATATTTCGCAACAA	GTGATACCCAGGGTGACGAT	(Hwang & Kim, 2024)
arnT	TGGCGCGAATAACTTTGGTG	ACGTTTATCGCGCCATAAGC	This study
arnE	AACGGCGCAAACATATCGTG	CTTAACATCGGGTAGGCAATGC	This study
ugd	TGCGTGAAGGTAAAGCGTTG	ATTCGGGCAAAACGTTCTGC	This study
eptB	TCCGCAGCGCACATTAAAAG	AAGCAATGTTGTCCGCCATC	This study
mdtG	GTCACCCTGTGAAAATGACACC	AAGGGCATTACCAGACTGAAGG	This study
acrZ	TGGGTCTGATTTACGGTCTTGG	TGATTTTGTCCGGGCTGGTC	(Hwang & Kim, 2024)
mdtE	AATGTCACCTCGCCGATTAC	CACTGGCGACCTCTTCTTTC	(Hwang & Kim, 2024)
16S rRNA	CGTGCTACAATGGCGCATAC	CTCCAATCCGGACTACGACG	(Bliven, 2015)

The thermal cycling protocol was as follows: initial denaturation for 10 min at 95 °C followed by 60 cycles of 15 s at 95 °C, 30 s at 60 °C, and 30 s at 72 °C. The fluorescence signal was measured at the end of each extension step at 72 °C. After the amplification step, melting curve analysis, with a temperature gradient of 0.1 °C/s from 60 to 95 °C, was performed to confirm that only the specific products were amplified. Finally, the samples were cooled to 20 °C for 10 s on a PikoReal 96 real-time PCR system (Thermo Fisher Scientific).

Table S2. List of top 50 differentially expressed genes (DEGs).

Rank	Gene	Product	Log_2 fold change	FDR*
up				
1	*ndk*	Nucleoside diphosphate kinase	8.85	9.22E-284
2		IS3 family transposase ISSfl10	7.92	2.80E-103
3		ISL3 family transposase ISEc38	7.82	9.23E-271
4	*ulaD*	3-keto-L-gulonate-6-phosphate decarboxylase UlaD	7.75	5.61E-136
5		hypothetical protein	7.29	7.34E-100
6	*ulaC*	Ascorbate-specific PTS system EIIA component	7.06	6.73E-14
7		hypothetical protein	6.51	0.00E+00
8		hypothetical protein	5.44	1.54E-119
9		hypothetical protein	5.38	2.02E-17
10		hypothetical protein	4.82	2.08E-120
11		hypothetical protein	4.79	0.00E+00
12	*yfbM*	Protein YfbM	4.60	2.87E-03
13		hypothetical protein	4.58	5.36E-50
14	*purE*	N5-carboxyaminoimidazole ribonucleotide mutase	4.40	1.18E-02
15	*yurK*	putative HTH-type transcriptional regulator YurK	4.36	6.59E-03
16	*stpA*	DNA-binding protein StpA	4.35	0.00E+00
17		hypothetical protein	4.34	9.55E-03
18	*metJ*	Met repressor	4.24	2.88E-16
19	*malY*	Protein MalY	4.22	3.45E-115
20	*safA*	Two-component-system connector protein SafA	4.16	3.40E-02
21	*elaB*	Protein ElaB	4.14	2.59E-02
22		hypothetical protein	4.13	3.39E-34
23	*nuoI*	NADH-quinone oxidoreductase subunit I	4.13	4.04E-07
24		hypothetical protein	4.07	5.31E-119
25		putative protein	4.06	8.02E-205
26	*ybdZ*	Enterobactin biosynthesis protein YbdZ	3.97	2.45E-02
27	*qmcA*	Protein QmcA	3.96	4.96E-48
28		hypothetical protein	3.91	6.28E-106
29	*pbpC*	Penicillin-binding protein 1C	3.87	4.61E-76
30		hypothetical protein	3.87	2.72E-93
31	*speC*	Constitutive ornithine decarboxylase	3.85	2.90E-02
32	*edd*	Phosphogluconate dehydratase	3.81	1.21E-15
33		hypothetical protein	3.77	1.43E-148
34	*glnK*	Nitrogen regulatory protein P-II 2	3.74	3.93E-02
35	*lgoT*	putative L-galactonate transporter	3.72	1.10E-123
36	*gltC*	HTH-type transcriptional regulator GltC	3.72	4.02E-60
37		hypothetical protein	3.71	4.23E-02

38	*pbpC*	Penicillin-binding protein 1C	3.71	1.39E-207
39	*recD2*	ATP-dependent RecD-like DNA helicase	3.71	0.00E+00
40	*yhaH*	Inner membrane protein YhaH	3.65	9.57E-36
41		hypothetical protein	3.64	5.23E-25
42		hypothetical protein	3.60	2.61E-13
43	*fixX_2*	Ferredoxin-like protein FixX	3.59	4.91E-02
44	*lolD*	Lipoprotein-releasing system ATP-binding protein LolD	3.55	5.79E-05
45	*noc*	Nucleoid occlusion protein	3.53	5.03E-80
46		hypothetical protein	3.51	5.86E-04
47	*viaA*	Protein ViaA	3.49	1.41E-276
48	*betA*	Oxygen-dependent choline dehydrogenase	3.49	8.06E-71
49	*yibH*	Inner membrane protein YibH	3.42	1.04E-37
50	*yeiR*	Zinc-binding GTPase YeiR	3.38	5.67E-67
down				
1		hypothetical protein	-16.25	0.00E+00
2	*iucD*	L-lysine N6-monooxygenase	-14.95	0.00E+00
3		hypothetical protein	-14.56	0.00E+00
4		IS91 family transposase ISSbo1	-14.49	0.00E+00
5		IS66 family transposase IS679	-14.40	0.00E+00
6		hypothetical protein	-14.39	0.00E+00
7		IS91 family transposase ISVsa3	-14.26	0.00E+00
8	*intQ*	Putative defective protein IntQ	-14.21	0.00E+00
9	*ipaA*	Invasin IpaA	-14.16	0.00E+00
10		IS66 family transposase ISCro1	-13.98	0.00E+00
11		putative E3 ubiquitin-protein ligase ipaH4.5	-13.94	0.00E+00
12		hypothetical protein	-13.87	0.00E+00
13		IS4 family transposase ISSfl1	-13.83	0.00E+00
14		putative E3 ubiquitin-protein ligase ipaH4.5	-13.80	0.00E+00
15		IS91 family transposase ISSbo1	-13.75	0.00E+00
16		IS4 family transposase ISEc60	-13.64	0.00E+00
17		IS4 family transposase ISEc60	-13.64	0.00E+00
18	*recB*	RecBCD enzyme subunit RecB	-13.61	0.00E+00
19		hypothetical protein	-13.55	0.00E+00
20		hypothetical protein	-13.47	0.00E+00
21		hypothetical protein	-13.46	0.00E+00
22		N-acetyldiaminopimelate deacetylase	-13.39	0.00E+00
23	*sepA*	Serine protease SepA autotransporter	-13.28	0.00E+00
24		hypothetical protein	-13.28	0.00E+00
25		IS91 family transposase ISSbo1	-13.28	0.00E+00
26		IS3 family transposase IS629	-13.08	0.00E+00

27	*iucC*	Aerobactin synthase	-13.07	0.00E+00
28		hypothetical protein	-13.05	0.00E+00
29		IS630 family transposase IS630	-12.91	1.91E-149
30		hypothetical protein	-12.84	0.00E+00
31		hypothetical protein	-12.82	0.00E+00
32	*ipaD*	Invasin IpaD	-12.74	0.00E+00
33		putative autotransporter	-12.55	0.00E+00
34		IS21 family transposase ISEc10	-12.45	0.00E+00
35		IS91 family transposase ISEc37	-12.44	0.00E+00
36		IS4 family transposase ISEc60	-12.32	7.30E-281
37		IS4 family transposase ISEc60	-12.32	7.30E-281
38	*iutA*	Ferric aerobactin receptor	-12.24	0.00E+00
39		hypothetical protein	-12.24	0.00E+00
40		IS3 family transposase IS3H	-12.23	0.00E+00
41		hypothetical protein	-12.18	0.00E+00
42		IS630 family transposase IS630	-12.15	0.00E+00
43		IS630 family transposase IS630	-12.15	0.00E+00
44		IS630 family transposase IS630	-12.15	0.00E+00
45		IS630 family transposase IS630	-12.15	0.00E+00
46		hypothetical protein	-12.13	0.00E+00
47		hypothetical protein	-12.10	0.00E+00
48	*repA*	Replication initiation protein	-12.09	0.00E+00
49	*parM*	Plasmid segregation protein ParM	-12.09	0.00E+00
50		hypothetical protein	-12.06	1.06E-260

*: False Discovery Rate

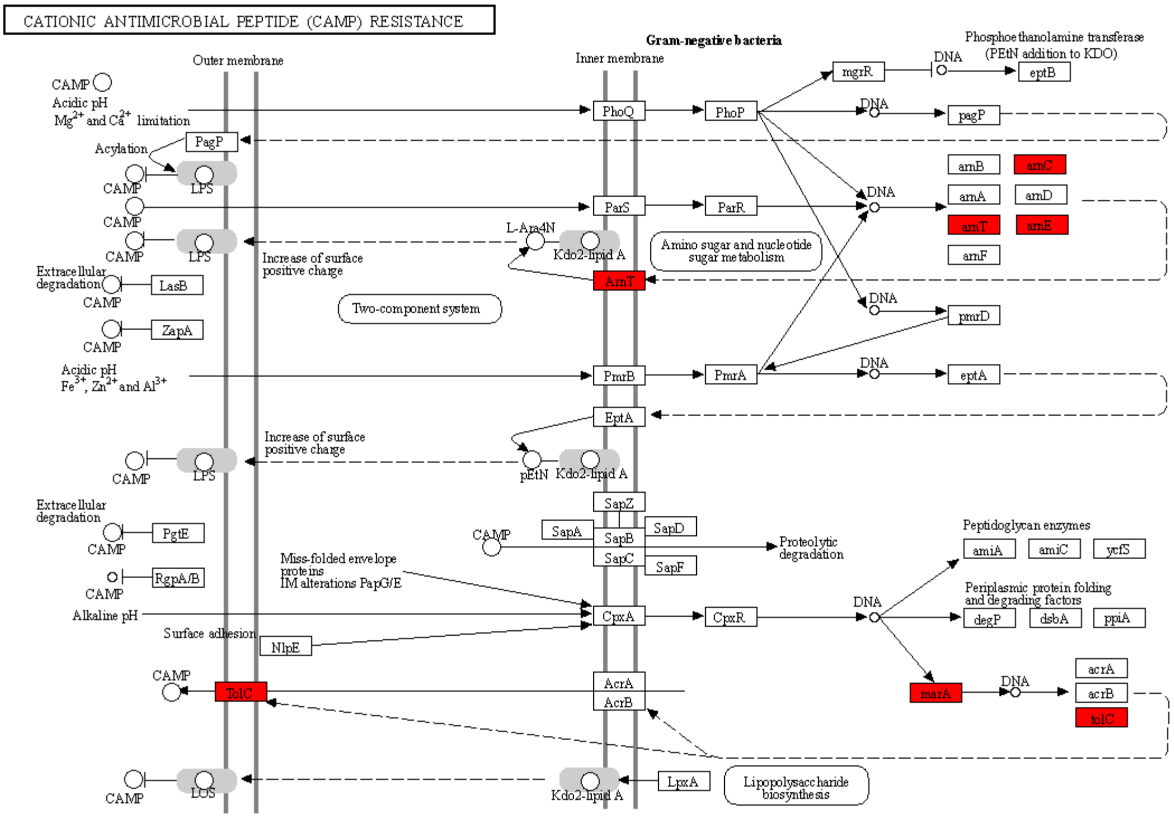

Figure S1. Identified several upregulated genes associated with cationic antimicrobial peptide resistance among KEGG pathways.

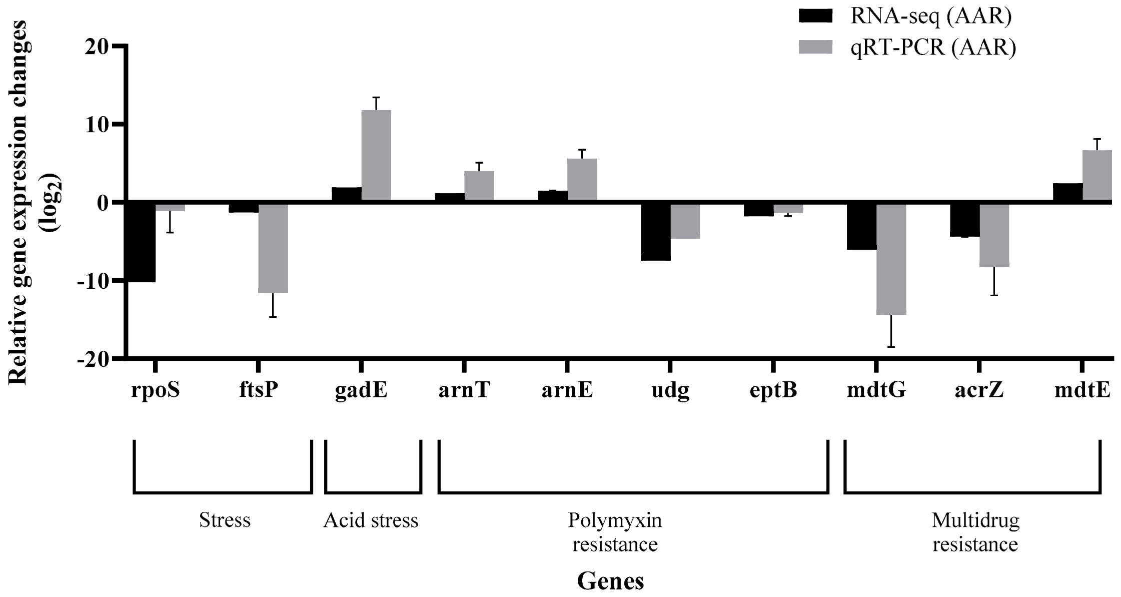

Figure S2. Validation of RNA-seq results via qRT-PCR. Expression level comparison of 10 selected DEGs between RNA-Seq and qRT-PCR.
